# Impact of a post‐donation hemoglobin testing strategy on efficiency and safety of whole blood donation in England: A modeling study

**DOI:** 10.1111/trf.17277

**Published:** 2023-02-16

**Authors:** Lois G. Kim, Thomas Bolton, Michael J. Sweeting, Steven Bell, Sarah Fahle, Amy McMahon, Matthew Walker, Eamonn Ferguson, Gail Miflin, David J. Roberts, Emanuele Di Angelantonio, Angela M. Wood

**Affiliations:** ^1^ Blood and Transplant Research Unit in Donor Health and Behaviour Cambridge UK; ^2^ Dept of Public Health & Primary Care University of Cambridge Cambridge UK; ^3^ BHF Data Science Centre Health Data Research UK; ^4^ Dept of Health Sciences University of Leicester Leicester UK; ^5^ Dept of Clinical Neurosciences University of Cambridge Cambridge UK; ^6^ School of Psychology University of Nottingham Nottingham UK; ^7^ NHS Blood & Transplant Bristol UK; ^8^ NHS Blood & Transplant John Radcliffe Hospital Oxford UK; ^9^ Radcliffe Dept of Medicine University of Oxford Oxford UK; ^10^ British Heart Foundation Centre of Research Excellence University of Cambridge Cambridge UK; ^11^ Health Data Research UK Cambridge, Wellcome Genome Campus and University of Cambridge Cambridge UK; ^12^ Health Data Science Research Centre Human Technopole Milan Italy; ^13^ Cambridge Centre of Artificial Intelligence in Medicine Cambridge UK

**Keywords:** blood donation, low hemoglobin deferral, post‐donation testing, simulation modeling

## Abstract

**Background:**

Deferrals due to low hemoglobin are time‐consuming and costly for blood donors and donation services. Furthermore, accepting donations from those with low hemoglobin could represent a significant safety issue. One approach to reduce them is to use hemoglobin concentration alongside donor characteristics to inform personalized inter‐donation intervals.

**Study Design and Methods:**

We used data from 17,308 donors to inform a discrete event simulation model comparing personalized inter‐donation intervals using “post‐donation” testing (i.e., estimating current hemoglobin from that measured by a hematology analyzer at last donation) versus the current approach in England (i.e., pre‐donation testing with fixed intervals of 12‐weeks for men and 16‐weeks for women). We reported the impact on total donations, low hemoglobin deferrals, inappropriate bleeds, and blood service costs. Personalized inter‐donation intervals were defined using mixed‐effects modeling to estimate hemoglobin trajectories and probability of crossing hemoglobin donation thresholds.

**Results:**

The model had generally good internal validation, with predicted events similar to those observed. Over 1 year, a personalized strategy requiring ≥90% probability of being over the hemoglobin threshold, minimized adverse events (low hemoglobin deferrals and inappropriate bleeds) in both sexes and costs in women. Donations per adverse event improved from 3.4 (95% uncertainty interval 2.8, 3.7) under the current strategy to 14.8 (11.6, 19.2) in women, and from 7.1 (6.1, 8.5) to 26.9 (20.8, 42.6) in men. In comparison, a strategy incorporating early returns for those with high certainty of being over the threshold maximized total donations in both men and women, but was less favorable in terms of adverse events, with 8.4 donations per adverse event in women (7.0, 10,1) and 14.8 (12.1, 21.0) in men.

**Discussion:**

Personalized inter‐donation intervals using post‐donation testing combined with modeling of hemoglobin trajectories can help reduce deferrals, inappropriate bleeds, and costs.

## 1 INTRODUCTION

Deferral of blood donors is an undesirable outcome for both the donor and blood donation service. Deferrals due to low hemoglobin occur in around 4–9% of blood donor visits, compared to approximately 4% that are deferred for other reasons (such as recent travel or illness), though this differs for new and returning donors and also depends on a wide range of factors including hemoglobin cut‐off for donation.^1^, ^2^, ^3^ Donors who are deferred, including those deferred for low hemoglobin, have lower rates of return.^4^, ^5^ Such deferrals also carry a cost to both the blood service^6^ and to the individual donor. Previous research has demonstrated that deferral for low hemoglobin^7^, ^8^, ^9^ and hemoglobin recovery^9^, ^10^ are associated with a number of factors, including age, ethnicity, length of inter‐donation interval, and hemoglobin level at the last donation. Therefore, one potential approach to reducing deferrals is to implement a post‐donation testing strategy, using a donor's hemoglobin level at the previous donation to calculate a personalized interval that provides some certainty that the donor will be eligible to donate. This approach also aims to identify donors most at risk of deferral and re‐invite them with a longer inter‐donation interval. Conversely, it may also be possible in principle to safely re‐invite some donors, especially those with high‐demand blood groups (e.g., O negative and O positive), to donate more frequently in the short‐term using a shorter interval in times of blood shortages. Such a strategy must be considered in parallel with donor safety, particularly the issue of inappropriate bleeds in donors below the regulatory threshold (which may occur as a result of measurement error in on‐site hemoglobin testing).

In this study, we use discrete event simulation (DES) modeling^11^ to estimate key clinical and cost‐to‐blood‐service outcomes for a hypothetical donor population in England under a range of personalized inter‐donation interval strategies aimed at reducing low hemoglobin deferrals. Personalized intervals for this population are determined by modeling post‐donation hemoglobin recovery trajectories based on data from the COMPARE study, a large diagnostic accuracy study of blood donors in England, originally designed to compare different methods to measure hemoglobin concentrations in whole blood donors.^12^

## 2 METHODS

A simulation model was developed to assess four different invitation strategies (see Table 1), together with the current strategy. The strategies are described below, followed by details of the DES model structure and parameterization.

**TABLE 1:** Modeled donor recall strategies.

Strategy type	Model name	Minimum recall interval (weeks; f,m)	Minimum probability over threshold at recall	Probability range for on‐site test
Fixed recall	(A) Current UK strategy	16, 12	‐	‐
Post‐donation testing	(B) Medium certainty	16, 12	0.7	‐
	(C) High certainty	16, 12	0.9	‐
	(D) High certainty, permit early recall	12, 8	0.9	‐
Post‐donation testing & on‐site testing	(E) High certainty, on‐site test for medium certainty	16, 12	0.7	0.7–0.9

### 2.1 Model strategies

#### 2.1.1 Current strategy

**FIGURE 1:**
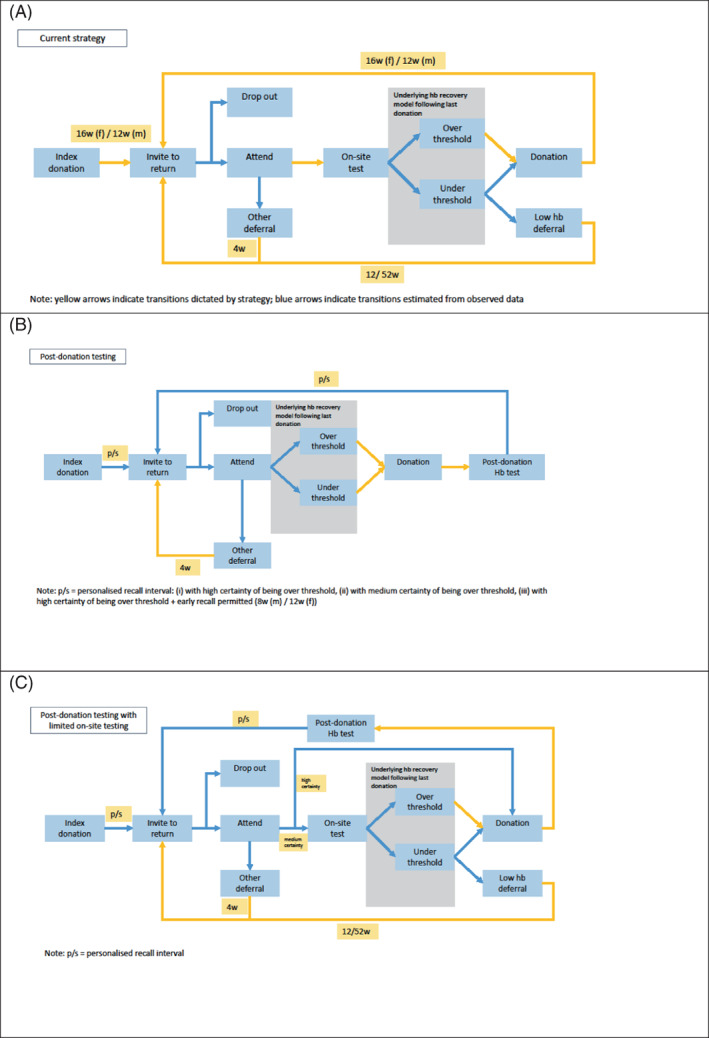
(A) Current strategy, (B) post‐donation testing strategies, (C) post‐donation testing with limited on‐site testing. [Color figure can be viewed at wileyonlinelibrary.com]

Under the current strategy (Strategy A; Figure 1A), individuals are invited to return a minimum of 12 weeks (men) or 16 weeks (women) after each donation, up to a maximum of four times per year. Donors can choose to return at any time after this interval, or may stop donating altogether (donor lapse or withdrawal). Amongst those returning, some are deferred on arrival for a range of reasons unrelated to hemoglobin (including other medical reasons, recent travel, or administrative reasons). As in the strategy employed at the time of the COMPARE study, the remaining eligible donors are screened for hemoglobin level estimated using a gravimetry/venous HemoCue approach (i.e., copper sulphate gravimetric test carried out on finger‐prick capillary blood, followed by spectrophotometry [HemoCue AB, Ängelholm, Sweden] on venous blood for those failing gravimetry).^13^

#### 2.1.2 Post‐donation testing

In addition to the current UK strategy, we model three strategies based on post‐donation testing (Figure 1B). In these strategies, the previous single hemoglobin measurement measured by a hematology analyzer is used to calculate the probability of the individual's hemoglobin being over the threshold level (≥135 g/L for men, ≥125 g/L for women) over time and thus inform a personalized inter‐donation interval. The three modeled strategies differ in the level of certainty that the donor is over the threshold to donate at the point of re‐invitation and/or shortest permitted interval:

Strategy B: medium (70%) certainty with minimum 16‐week return for women/12‐weeks for men, Strategy C: high (90%) certainty with minimum 16‐week return for women/12‐weeks for men, and Strategy D: high (90%) certainty with minimum 12‐week return for women/8‐weeks for men (i.e., earlier than currently allowed).

Strategy D incorporates some returns that are earlier than regulations currently permit as an exploratory investigation of a novel, personalized approach to earlier returns. In this strategy, those invited for early return visits are a small subset with predicted rapid hemoglobin recovery and/or very high hemoglobin levels, since they must also meet the high‐certainty criterion at this early time‐point. Under all three strategies, there is no on‐site testing, and all those returning to the donor center donate (minus a small proportion deferred for other reasons). The donor's blood is subsequently tested post‐donation using a hematology analyzer (Sysmex XN‐2000 hematology analyzer), with the resultant accurate hemoglobin level used to inform further recovery modeling and personalized intervals.

#### 2.1.3 Post‐donation testing with limited on‐site testing

The final strategy (Strategy E; Figure 1C) also uses post‐donation testing to inform inter‐donation intervals with medium or high certainty of being over the threshold, alongside limited on‐site testing using portable hemoglobinometry (HemoCue AB, Ängelholm, Sweden) for donors with medium certainty. Donors under the threshold at on‐site testing are re‐invited after the standard deferral period.

### 2.2 Model parameterization

The DES tracks outcomes for each of the strategies applied over a one‐year period. Transitions (indicated with blue arrows in Figure 1) are parameterized using data from the COMPARE study.

#### 2.2.1 COMPARE study

The COMPARE study (ISRCTN 90871183) recruited donors in England between February 2016 and March 2017 with the aim to compare hemoglobin measurements taken using different methods in the same participants. Donors were eligible if they were aged 18+, fulfilled routine criteria for donation, had an email address and internet access to respond to questionnaires, and were willing to undergo additional hemoglobin measurements. The recruited donor population was broadly similar to the general blood donor population of England, notwithstanding some minor differences in age, sex, ethnicity, and donation history. Full details of the original study are available elsewhere.^12^ Here, these data serve as a returning donor population in whom baseline characteristics and repeat accurate hemoglobin levels are available.

We use data from stage 1 donors in COMPARE with non‐missing blood group, ethnicity, and index donation hemoglobin (i.e., initial donation at baseline) to inform estimation of parameters in the DES: a total of 9360 women and 7948 men. Baseline and return visit characteristics of this subsample are provided in Suppl S1. 8441 (90%) women and 7421 (93%) men returned within a year, reflecting the commitment of those who consented to the study. Of these, 78% of women and 80% of men had known hemoglobin levels at the second visit for use in parameter estimation.

#### 2.2.2 Parameter estimation

Using data from the COMPARE study, estimates were obtained for time to attendance following invitation to donate as well as parameters relating to non‐hemoglobin deferrals and non‐attendance (Suppl S2). We undertook statistical modeling of the post‐donation hemoglobin recovery over time, adjusted for baseline age, blood group, and ethnicity, and stratified by sex (Suppl S3). This model is based on a maximum of two donations in the COMPARE study population and is consequently limited to assuming a common recovery rate over time, though allows for differences in index donation hemoglobin. These estimates are applied in the DES to all donations taking place over a 1‐year period. Predictions from the hemoglobin recovery model after each donation in the DES are then used to underpin the personalized inter‐donation intervals in the post‐donation strategies modeled in the DES by identifying the time at which a donor's hemoglobin is estimated to be over the donation threshold (125 g/L for women, 135 g/L for men) (Supp S3.2). Since these predicted hemoglobin levels are not known with absolute certainty, variations on this strategy employ different minimum probabilities that the estimated hemoglobin is over this threshold; the time at which this minimum probability is met is then used as the donor's inter‐donation interval.

For donors undergoing on‐site testing in the DES (in both the current strategy (A) and the post‐donation testing plus on‐site testing strategy (E)), the donor's modeled hemoglobin value is used to indicate whether they pass or fail this test, that is, those with modeled hemoglobin levels above the donation threshold are assumed to pass the test and donate. A proportion of those with modeled hemoglobin levels below the threshold may also pass the test (i.e., an inappropriate bleed) whilst the remainder fail the test (i.e., a low hemoglobin deferral). This reflects the observed pattern in the COMPARE study (Suppl S2).

As under current policy, individuals deferred for low hemoglobin in the DES are re‐invited to donate after 12 weeks (or 52 weeks if the modeled hemoglobin is very low, defined as <115 g/L for women and <125 g/L for men).Those deferred for other reasons are re‐invited to donate after 4 weeks, reflecting the most common deferral period in COMPARE, which ranged from 1 to 180 days.

#### 2.2.3 Costs

Costs are considered from the perspective of the blood service (Table 2) and are applied to hemoglobin tests and donations, including invitation costs. Deferrals are also costed, in order to account for tests, staff time, and associated downstream care costs for these donors.

**TABLE 2:** Unit costs to the blood service applied in the discrete event simulation (DES) model.

Costed event	Cost (£, 2019)	Source
On‐site test (venous Hemocue)^a^	£1.08	Grieve et al^e^
Off‐site test (Sysmex)^b^	£0.79	R Blanco^f^ (personal communication, Aug 2021)
Donation^c^	£26.49	Grieve et al (Appendix 18)^e^
Deferral (low hemoglobin)^d^	£9.21	Grieve et al (Appendix 18)^e^
Deferral (other)	£0.97	Grieve et al^e^

^a^ Includes capital outlay for machine, lifetime of machine, and consumables; applied to a proportion of donors who fail copper sulphate test.

^b^ Includes capital outlay for machine, lifetime of machine, consumables, and staff costs (band 4 SHTO).

^c^ For static venue with zero venue costs; includes staff costs and 5.3 × appointment letters per donation, copper sulphate test and consumables relating to donation.

^d^ Includes copper sulphate test, venous Hemocue, staff costs, and down‐stream health‐care costs.

^e^ Grieve R, Willis S, De Corte K, et al. Options for possible changes to the blood donation service: health economics modeling. *Health Services and Delivery Research* 2018; **6(40)**.

^f^ Component Development Laboratory, NHS Blood & Transplant, Cambridge.

### 2.3 Model implementation

The DES draws 10,000 hypothetical men or 10,000 hypothetical women with replacement from the COMPARE study population (this ensures correlation of baseline characteristics is retained in the simulated population) and tracking outcomes over 1 year. Key events extracted for each individual and summarized over the whole population are: number of donations (total and number under the donation threshold), number of low hemoglobin deferrals, and costs. For the purposes of this work, adverse events are defined as low hemoglobin deferrals and donations under the recommended threshold combined. Uncertainty intervals are obtained by drawing from the joint distribution of all data‐informed parameter values (1000 draws of 1000 individuals).

### 2.4 Internal validation

An internal validation comparing the DES results with observed data from COMPARE was carried out based on first return only. This reflects the information available in COMPARE, with model outputs restricted to first returns to provide comparable results. To accurately reflect observed return times in COMPARE (as opposed to current policy), the internal validation employed a modification that permitted returns in women as early as 12 weeks. This was necessary because in COMPARE, 18% of female donors returned between 12 and 16 weeks post‐index donation. Women were advised not to return earlier than 16 weeks, but a small number opted to return as early as 12 weeks, as permitted under regulations. The booking system has been revised since this study was conducted and women are no longer able to book earlier than 16 weeks after a donation.

Modeling was performed using R Version 4.0.3 (2020‐10‐10).

## 3 RESULTS

### 3.1 Internal validation

Numbers of predicted key events from the DES are broadly similar to observed events from COMPARE, scaled to 1000 men and 1000 women (Table 3). Specifically, numbers of donors dropping out and returning, deferred for reasons other than low hemoglobin, and over threshold donations are very similar. However, the number of inappropriate bleeds is under‐estimated in the model in both women (114 vs. 125, −9%) and men (51 vs. 68, −25%). Low hemoglobin deferrals are also under‐predicted, particularly in men (21 vs. 27, −22%). These discrepancies arise in part because the underlying model for hemoglobin fits less well at the most extreme values of hemoglobin at recall and because the threshold for donation in men is further into the tail of the hemoglobin distribution than in women (Suppl S3.1, Figure S2).

**TABLE 3:** Internal validation—numbers of observed and predicted events (per 1000 donors).

Strategy	Dropout	Returned	Other deferrals	Over threshold donations	Adverse events		Donations per adverse event
					Under threshold donations	Low hemoglobin deferrals	
**Women**							
Observed^a^	98	902	49	658 (518, 685)	125 (99, 266)	69	4.7
Predicted^b^	99	893	48	665	114	66	4.3
*Difference*	*+1%*	*−1%*	*−2%*	*+1% (−3%, +28%)*	*−9% (−57%, +15%)*	*−4%*	*‐*
**Men**							
Observed^a^	66	934	37	801 (649, 814)	68 (55, 220)	27	10.6
Predicted^b^	66	927	39	816	51	21	12.1
*Difference*	*0%*	*−1%*	*+5%*	*+2% (0%, +26%)*	*−25% (−7%, −77%)*	*−22%*	*‐*

^a^ Exact numbers over/under threshold in COMPARE are not known because 22% of female donating returners and 20% of male donating returners in the study have unknown hemoglobin values at return. The main figure given assumes the same proportions over/under the threshold in those with unknown hemoglobin as observed in those with known hemoglobin. Figures in parentheses show the potential range of values at the extremes of this assumption, that is, if all those with unknown hemoglobin are either all under or all over the threshold.

^b^ Current strategy model, with modification of return time for women for validation purposes (see text for details).

### 3.2 Results from the discrete event simulation model

Table 4 summarizes re‐invitation times under each strategy. The results show pronounced benefits of personalized inter‐donation intervals in women. For example, whilst the vast majority (98%) of men are re‐invited at the minimum time under all strategies, in women, 12–16% (depending on strategy) are re‐invited later, including 7–10% at 24+ weeks post‐donation. This reflects those women in whom low hemoglobin deferral is averted under a personalized strategy. That the minimum inter‐donation intervals is applied to the majority of men is largely because most of the variation in hemoglobin at the re‐invitation is accounted for by donor characteristics rather than time to return (Table S5).

**TABLE 4:** Minimum inter‐donation intervals during first year following index donation, by strategy.

	Strategy				
	A: Current	B: Medium certainty	C: High certainty	D: High certainty, early recall	E: High certainty, on‐site test for medium certainty
**Women**					
Total return visit invitations^a^	2079	1688	1498	1936	1681
*Of which*:					
12 weeks	‐		‐	85.0%	‐
13–15 weeks	‐		‐	1.8%	‐
16 weeks	100%	88.2%	84.9%	1.5%	88.0%
17–23 weeks	‐	4.0%	4.9%	4.3%	4.2%
24+ weeks	‐	7.8%	10.3%	7.4%	7.8%
**Men**					
Total return visit invitations^a^	2495	2267	2115	2792	2282
*Of which*:					
8 weeks	‐		‐	97.8%	‐
9–11 weeks	‐		‐	0.5%	‐
12 weeks	100%	98.4%	98.0%	0%	98.4%
13–17 weeks	‐	0.6%	0.8%	0.6%	0.6%
18+ weeks	‐	1.0%	1.2%	1.1%	1.0%

*Note*: simulation run on 10,000 hypothetical donors; results here scaled to *n* = 1000 and reported for a 1‐year period.

^a^ Includes all post‐donation recall invitations <1 year after index donation, even if attendance occurs beyond 1 year. Excludes recall invitations following deferral for any reason.

Number of donations (per year), inappropriate bleeds, low hemoglobin deferrals, and costs by strategy are shown in Table 5. These results reflect the numbers of events amongst a hypothetical population of 1000 donors donating at the start of the year, summarizing outcomes from subsequent visits (thus incorporating non‐attendance) over the course of the following year. There is no single strategy that maximizes total donations and also minimizes adverse events and costs. Therefore, the preferred strategy will necessarily depend on the perceived preference weighting between these elements.

**TABLE 5:** Key events and costs for alternative strategies, all recalls in 1 year.

Strategy	Adverse events (% of return visits)		Total donations excl index donation	Donations per adverse event	Mean cost per donation^a^
	Inappropriate bleeds	Low Hb deferrals			
**Women**					
A: Current	16.0% (14.2%, 18.8%)	9.3% (8.1%, 11.6%)	1608 (1539, 1653)	3.4 (2.8, 3.7)	£27.64 (£27.45, £27.87)
B: Medium certainty	10.9% (9.2%, 13.0%)	‐	1385 (1279, 1450)	8.7 (7.3, 10.3)	£27.37 (£27.36, £27.38)
C: High certainty	6.4% (5.0%, 8.1%)	‐	1223 (1100, 1319)	14.8 (11.6, 19.2)	£27.37 (£27.36, £27.38)
D: High certainty, permit early recall	11.3% (9.4%, 13.4%)	‐	1613 (1502, 1716)	8.4 (7.0, 10.1)	£27.37 (£27.36, £27.38)
E: High certainty, on‐site test for medium certainty	8.2% (7.0%, 10.0%)	3.3% (2.2%, 4.4%)	1348 (1256, 1421)	7.6 (6.6, 9.4)	£27.73 (£27.61, £27.86)
**Men**					
A: Current	9.1% (7.5%, 11.0%)	3.8% (2.8, 4.8%)	1991 (1931, 2057)	7.1 (6.1, 8.5)	£26.93 (£26.84, £27.05)
B: Medium certainty	6.0% (4.6%, 7.3%)	‐	1864 (1787, 1936)	16.0 (13.2, 20.8)	£27.35 (£27.34, £27.36)
C: High certainty	3.6% (2.2%, 4.6%)	‐	1730 (1653, 1833)	26.9 (20.8, 42.6)	£27.35 (£27.34, £27.36)
D: High certainty, permit early recall	6.5% (4.5%, 7.9%)	‐	2327 (2222, 2416)	14.8 (12.1, 21.0)	£27.35 (£27.34, £27.36)
E: High certainty, on‐site test for medium certainty	5.4% (4.1%, 6.4%)	1.1% (0.3%, 1.6%)	1847 (1777, 1922)	14.7 (12.5, 20.7)	£27.46 (£27.38, £27.53)

*Note*:Results shown in parentheses indicate 95% uncertainty intervals derived from 1000 bootstrap samples of parameter values informing the model. Each bootstrap sample models 1000 individuals, with results scaled to *n* = 1000. Grayed out rows indicate strategies where alternatives offer both higher total donations and higher donations per adverse event at a cheaper/equal cost.

a Point estimate given as median from bootstrap samples.

Donations per adverse event are maximized under strategy C (high certainty without early recall), with an estimated 14.8 donations/adverse event in women (95% uncertainty interval 11.6, 19.2) and 26.9 donations/adverse event in men (95% UI 20.8, 42.6). This strategy also minimizes costs in women at £27.37/donation. For both men and women, strategy D (high certainty with early recall) maximizes donations whilst also minimizing costs in women at £27.37/donation in women (Table 5). In men, costs are minimized at £26.93/donation under the current strategy (A), but this strategy is least favorable in terms of adverse events, at 7.1 donations per adverse event (95% UI 6.1, 8.5). The current strategy also has the least favorable donations per adverse events in women. In both sexes, strategy E (high certainty with on‐site test for medium certainty) is dominated: better outcomes (higher total donations and higher donations/adverse event) at lower cost are possible with alternative strategies.

## 4 DISCUSSION

This modeling study explored the impact of various post‐donation testing strategies combined with personalized inter‐donation intervals on key donor outcomes, including cost per donation, total number of donations, and donations per adverse event (low hemoglobin deferrals and inappropriate bleeds). Data from COMPARE were used to inform and validate the model. The model can be used to explore different strategies for hemoglobin testing in donors and some possible approaches are compared in this analysis. A strategy of re‐inviting donors with a ≥90% probability of future hemoglobin level (based on last previous measurement) being above the donation threshold yielded the highest number of donations per adverse event in both sexes and also had the lowest cost per donation in women. This represents an estimated reduction of 111,000 low hemoglobin deferrals and 128,000 inappropriate bleeds per annum, based on 1.4 m blood donations per annum and 51% donations from females.^14^ This strategy also uses only post‐donation testing for hemoglobin (compared to the current strategy of on‐site testing only, or alternative strategies using a combination of post‐donation and on‐site testing), thus improving operational efficiency and, in women, reducing costs. Furthermore since adverse events disproportionately occur in women, this strategy also goes some way to redressing this inequality as inappropriate bleeds are minimized and low hemoglobin deferrals are eliminated. The identification of post‐donation testing as a preferred strategy provides evidence‐based support for policies already in place in several European counties, including France, Belgium, and Denmark.^15^

A small number of other studies have explored alternative donor strategies.^16^, ^17^ Spencer *et al*.^17^ used simulation modeling to examine the potential impact of altering the inter‐donation intervals and/or reducing the threshold for donation on total number of donations. They concluded that lengthening inter‐donation intervals could substantially impact blood availability, and that whilst reductions in thresholds could increase stocks, strategies to combat iron deficiency anemia would also be needed. However, this study was limited to whole population strategies rather than our personalized approach, and did not consider the possibility of post‐donation testing. Furthermore, costs and under‐threshold donations were not reported. The INTERVAL trial was a randomized trial aimed at assessing the effect of different fixed inter‐donation intervals on blood supply and donor health, providing evidence for the safety of inter‐donation intervals ±2 weeks compared to the UK status quo.^6^, ^18^ The COMPARE study^12^ itself considered the potential impact of a last‐observation carried forward post‐donation approach, though these results are not directly comparable to ours since they focus on only the first recall visit. Drawing on work examining the plasma donor's perception concerning the frequency of donation indicates that these donors would be willing to donate at a higher frequency, but the main concern with doing so was the implication this may have for their health.^19^, ^20^, ^21^ As such, evidence that allows for a better understanding of how post‐donation testing can identify the optimal interval window (including short intervals) to protect donor health would be valuable to developing donor communications and policy. Here, we build on this work to personalize the use of reduced (or lengthened) inter‐donation intervals based on a post‐donation testing strategy over a one‐year period.

### 4.1 Strengths and limitations

We have assessed population‐level outcomes, including costs, relevant to England's blood service for a range of pragmatic post‐donation testing strategies combined with personalized inter‐donation intervals. Some of the strategies considered incorporate a more personalized approach to inter‐donation intervals, utilizing routinely available donor characteristics such as age, blood group and ethnicity, to help inform optimal return times for donors. Data from a large observational study of blood donors were used to estimate event rates and probabilities, with a mixed‐effects model of post‐donation hemoglobin to underpin the post‐donation and personalized inter‐donation strategies. Overall, the DES gave good internal validation, closely matching the COMPARE data for attendances, non‐hemoglobin‐related deferrals, and over threshold donations. Adverse events were also well‐matched in women, though there was some under‐estimation of these in men.

Our study also has a number of potential limitations. Firstly, with respect to the underlying hemoglobin model, the informing data are limited to a maximum of two measurements per donor in COMPARE (thus restricting the modeling to the assumption of a common rate of recovery over time) and we do not consider the possibility of an informative observation process of hemoglobin values at the return visit (i.e., that the hemoglobin levels of those not returning, or returning later than expected, may be associated with their true unobserved hemoglobin values). Secondly, we assume a linear hemoglobin recovery trajectory. However, explorations of model fit together with the focus on the middle (8+ weeks) part of the post‐donation period where the trajectory is broadly linear^22^ provide some confidence that this assumption is reasonable for the purposes of the modeling carried out here. Thirdly, we re‐applied the hemoglobin recovery model in the DES to multiple return visits over time (maximum of 6 in 1 year), even though we only observed one return visit in COMPARE; however, we do not extrapolate beyond 1 year of follow‐up in the DES model. Fourthly, we employed a limited set of predictors for both return time and hemoglobin based on their availability in donor records, but there may be other influences (e.g., frequency of mobile center area visits, season, lifestyle, and other biochemical and genetic factors) that could improve the prediction of these aspects of the model. For example, ferritin measures were not available in COMPARE, and have thus not been considered here, but may offer a useful addition to identifying those donors most at risk of adverse events.^23^ A further simplification is that we also did not incorporate predictors into the parameter estimates for non‐hemoglobin deferral and dropout. Finally, following the results of COMPARE, some aspects of blood donation testing in England were altered from 2018 in order to address the high proportion of donations made in donors under the threshold for donation.^24^ Specifically, implementation of the initial copper sulphate gravimetric test was altered to improve accuracy, and the subsequent spectrophotometry test was switched to capillary (rather than venous) blood. Therefore, some parameter estimates relating to proportions deferred may not accurately reflect current English blood donation policy.

We have considered the potential benefits of alternative post‐donation strategies compared to the current on‐site testing and deferral approach. Although we have reported costs and adverse events alongside summarizing benefits, we have not incorporated any change in donor behavior in terms of unintended consequences that might arise as a result of adopting the post‐donation approach.^25^, ^26^ For example, some donors may prefer finger‐prick on‐site testing or the ability to book their next donation appointment at the end of the current session. Donors offered shortened donation intervals may feel pressured to donate whilst those offered lengthened donation intervals may become concerned about their health or suitability to donate. However, it is also possible that donors may welcome the personalization of return times and may value the reduced risk of deferral.^27^ Thus, it is important that unintended consequences are incorporated into a post‐implementation evaluation,^25^ and in particular that pre‐existing inequalities in donor populations are not exacerbated by any alterations to policy. To try and avoid unintended consequences, small‐scale studies (akin to phase I and II clinical trials) can be used,^28^ as well as developing communication strategies to frame any policy changes.

### 4.2 Conclusions

Adopting a re‐invitation policy that recommends longer inter‐donation intervals for those most at risk of deferral could substantially reduce low hemoglobin deferrals and below‐threshold bleeds. Additionally, implementing this through a modeling approach based on accurate post‐donation hemoglobin measurements eliminates the need for on‐site testing, offering benefits both in terms of time and cost to the blood service. Further studies, including blood donor involvement and engagement, and a small‐scale pilot, are now needed to confirm acceptability, feasibility, and safety in practice and to establish the requisite operational capacity.

## FUNDING INFORMATION

Funding for COMPARE was provided by NHSBT and the NIHR Blood and Transplant Research Unit (BTRU) in Donor Health and Genomics (NIHR BTRU‐2014–10,024). The academic coordinating center for COMPARE was supported by core funding from: NIHR BTRU, UK Medical Research Council (MR/L003120/1), British Heart Foundation (RG/13/13/30194; RG/18/13/33946) and the NIHR Cambridge Biomedical Research Centre (BRC‐1215‐20014). This work was supported by Health Data Research UK, which is funded by the UK Medical Research Council, Engineering and Physical Sciences Research Council, Economic and Social Research Council, Department of Health and Social Care (England), Chief Scientist Office of the Scottish Government Health and Social Care Directorates, Health and Social Care Research and Development Division (Welsh Government), Public Health Agency (Northern Ireland), British Heart Foundation and Wellcome. LK was funded by the NIHR BTRU in Donor Health and Genomics (NIHR BTRU‐2014‐10,024) and is funded by the NIHR BTRU in Donor Health and Behavior (NIHR203337). TB was funded by the NIHR BTRU in Donor Health and Genomics (NIHR BTRU‐2014‐10,024). SB was funded by the NIHR BTRU in Donor Health and Genomics (NIHR BTRU‐2014‐10,024). SF was funded by the NIHR BTRU in Donor Health and Genomics (NIHR BTRU‐2014‐10,024) and is funded by the NIHR BTRU in Donor Health and Behavior (NIHR203337). AM was funded by the NIHR BTRU in Donor Health and Genomics (NIHR BTRU‐2014‐10,024) and is funded by the NIHR BTRU in Donor Health and Behavior (NIHR203337). MW is funded by a BHF Chair award (CH/12/2/29428). AMW is part of the BigData@Heart Consortium, funded by the Innovative Medicines Initiative‐2 Joint Undertaking under grant agreement No 116074.

## CONFLICT OF INTEREST

Lois G. Kim, Thomas Bolton, Steven Bell, Sarah Fahle, Amy McMahon, Matthew Walker, Eamonn Ferguson, Gail Miflin, David J. Roberts, Emanuele Di Angelantonio and Angela M. Wood declare no conflict of interest. Michael J. Sweeting is a full‐time employee of AstraZeneca.

## Supporting information


**Data S1.** Supporting Information

## Data Availability

Individual‐level data from COMPARE is available to bona fide researchers upon reasonable request at https://www.comparestudy.org.uk/. All code is available at: https://github.com/drloiskim/blood_donation.git.
