# Supplementary material for: Impact of a post‐donation hemoglobin testing strategy on efficiency and safety of whole blood donation in England: A modeling study
Source: Transfusion. 2023 Feb 16;63(3):541–51. doi: 10.1111/trf.17277 (PMC10952564; doi:10.1111/trf.17277)

**Supplementary material**

**S1 COMPARE**

Data from the COMPARE study used for this work is summarised in Suppl Table 1. In COMPARE, the mean haemoglobin (Hb) at index visit was 135g/L (SD 10g/L) in women and 151g/L (SD 10g/L) in men. Information on the first follow-up visit within the subsequent year was also collected. In this period, 8441 (90%) women and 7421 (93%) men returned after a median inter-donation period of 17 weeks for both men and women. The mean haemoglobin at the follow-up visit was lower at 131g/L (SD 10g/L) in women and 147g/L (10g/L) in men, based on the 78% of women and 80% of men with known haemoglobin at the second visit (which includes both those who donated and those who were deferred).

**S2 Transition parameter estimation**

Estimates are obtained using a subset of the Stage 1 COMPARE population^12^ who were invited back to donate again during the study follow-up, with non-missing baseline haemoglobin (though return visit haemoglobin may be missing in those attending for a second donation) and baseline characteristics. Those with missing blood group (0.5%) or ethnicity (1%) were excluded from all analyses. No donors were missing age or number of low haemoglobin deferrals in the two years prior to baseline. Ethnicity is modelled as a binary variable (white v other), age as a categorical variable (<25, 25-34, 35-44, 45-54, 55-64, 65+), previous low haemoglobin deferrals in the 2 years prior to index donation (0, 1, 2+) as categorical and previous donations in the 2 years prior to index donation (0-2, 3-4, 5+) as categorical. Men and women are modelled separately.

S2.1 Time from recall to attendance

Modelling of return times is based on time from recall (e.g. 12 weeks) to attendance rather than time from index donation (i.e. 0 weeks) to next attendance. Flexible parametric rate modelling is used for returners, with adjustment for baseline covariables. Plots and the Akaike information criterion (AIC) are used to assess model fit (Suppl Fig 2, Suppl Table 2), with the 2-knot model (Suppl Table 3) providing a substantial improvement over the 0 (Weibull) and 1-knot models, particularly for men. The 2-knot model is applied in the discrete event simulation model (DES) for both men and women, with draws taken from the simulated distribution in hours after eligible to return, then re-converted back to weeks after simulation. The adjustment to time-scale was necessary to avoid errors arising from simulating values extremely close to zero weeks. Observed and simulated distributions of return donation visit times are summarised for women (Suppl Fig 3) and men (Suppl Fig 4). These models incorporate women attending earlier than the permitted interval as attending at the permitted interval (i.e. as early as possible, but not earlier than the permitted time). For validation purposes only, an additional model for women allowing returns as early as 12 weeks (i.e. as observed) is also fitted. An additional plot comparing distributions based on the validation model is given in Suppl Fig 5.

S2.2 Dropout, non-haemoglobin deferral, low haemoglobin deferral

The probabilities of non-return in the 1-year follow-up period, deferral for reasons other than low haemoglobin, and deferral for low haemoglobin (amongst those with haemoglobin below the threshold for donation at attendance) are estimated directly from the data, see Suppl Table 4.

Note that of those with known haemoglobin, over the threshold and not deferred for other reasons, 99.8% (4847/4859) of women and 99.9% (5160/5164) of men donate. The DES assumes that all donors above the threshold donate. However, amongst those with haemoglobin measured below the threshold only 37% of women and 29% of men were actually deferred.

S2.3 Uncertainty intervals

Uncertainty intervals for model outputs are obtained by taking 1000 draws from the joint distribution of parameter values, and running each for 1000 donors. For dropout, non-haemoglobin deferral and low haemoglobin, the mean and variance on which this draw is based are derived from a binomial distribution. For return time and haemoglobin, parameter coefficients and their corresponding variances are extracted from the models described in S2.1 and S3 respectively.

**S3 Haemoglobin modelling**

Haemoglobin recovery over time following initial donation is modelled using a linear mixed model, in order to incorporate measurements from both those with and without follow-up measurements. A visit indicator and time between visits are both included as predictors in the model, along with other baseline characteristics, and a random effect at the individual level. Since each individual has at most two observed haemoglobin measurements, this can be thought of as a common recovery rate (slope) over time for all individuals, with an individual-specific baseline measurement (intercept). Estimates from this modelling are given in Suppl Table 5. The random effects estimates (best linear unbiased predictors (BLUPs)) reflect the best-fit estimate of this baseline haemoglobin, after accounting for baseline characteristics and the recall visit measurement.

The model does not include previous donations or deferrals in the two years prior to baseline (for low haemoglobin or other reasons), as strictly these are time-varying covariables and without further information about the timing of these previous events it is not obvious how these might be updated in the DES.

*Note on use of BLUPs in DES model*

At the start of the DES model, a fixed effect and random effect (BLUP) are drawn for each modelled donor given their baseline characteristics. These are then used to calculate haemoglobin and its SE over time. On modelling haemoglobin recovery following subsequent donations, a new BLUP is calculated accordingly, maintaining the same SE from the mean as the original draw for this individual.

S3.1 Model fit

We investigated the possibility of non-linearity formally through fitting quadratic and cubic terms for inter-donation interval in the haemoglobin mixed model and through fitting fractional polynomial models. In women, there was no evidence that quadratic, cubic or any fractional polynomial terms improved the fit of the model over a linear model (all p>0.5). In contrast in men, there was strong evidence (p<0.001) that a cubic term improved the fit of the model, though the coefficients indicate a rapid increase in Hb in the later weeks rather than a plateau and as such are more likely to reflect informative return times than the underlying Hb recovery process. The fractional polynomial model identified log and -1/2 powers as providing the best fit (p=0.003 compared to linear model). However, from a practical perspective the predicted values were broadly similar to those from the linear model (Suppl Fig 1).

Observed versus predicted (fixed plus random effects) plots based on the linear mixed model for both women and men are shown in Suppl Fig 6. Visually these show reasonable agreement and offer some confidence in using a linear model for this part of the recovery process. However, note that whilst the misclassifications based on donation threshold in women largely cancel out (i.e. the number predicted to be under the threshold who are observed to be over the threshold is approximately equal to the number predicted to be over the threshold who are observed to be under it), in men there is a larger discrepancy, with a larger number predicted to be over the threshold who are observed to be under it. Although the underlying haemoglobin recovery may be non-linear, it is likely that a large part of this non-linearity lies in the early part of the recovery period immediately following donation^22^. Since the COMPARE study does not have return visits earlier than 12 weeks following the baseline donation, we are not attempting to model this part of the recovery. Further, none of the strategies propose recall earlier than 8 weeks, hence we are also not making inferences about the early part of the recovery. Finally, in only running the DES for one year, we are not extrapolating beyond the latest observed return time in the data.

3.2 Estimation of time of crossing the donation threshold in the DES

In order to estimate time to crossing the donation threshold for a given individual, visit, and required probability level (which is then used to inform eligibility for return under each strategy), a discrete grid of weeks from t=1 to 52 after a donation was created:

1. For each t, calculate Hb(t) using the linear predictor from the mixed model including the individual BLUP (and which includes the effect of t on Hb).

2. Look up the SE for (i) this particular combination of covariates and t from a dataset holding this information derived from the underlying mixed model and (ii) the BLUP.

3. Use calculated Hb(t) and SE(t) with pre-defined Hb threshold to calculate the probability that Hb(t) exceeds this threshold, under the assumption of normality.

4. Define the recall time as the minimum t at which this probability exceeds the uncertainty threshold for this strategy.

**References**

**Suppl Table 1 Data summary for COMPARE data**

|  | **Women** | **Men** |
| --- | --- | --- |
| N* | 9360 | 7948 |
|  |  |  |
| Mean Hb at index visit (SD), g/dl | 13.5 (1.0) | 15.1 (1.0) |
| N with return visit | 8441 (90%) | 7421 (93%) |
| Median return time (IQR), weeks | 17 (16, 20) | 17 (15, 19) |
| N with return Hb | 6547 (78% of returners) | 5946 (80% of returners) |
| Mean Hb at return visit (SD), g/dl | 13.1 (1.0) | 14.7 (1.0) |
|  |  |  |
| Age category (years) |  |  |
| <25 | 737 (8%) | 409 (5%) |
| 25 – 34 | 1368 (15%) | 853 (11%) |
| 35 – 44 | 1821 (19%) | 1161 (15%) |
| 45 – 54 | 2537 (27%) | 2271 (29%) |
| 55 – 64 | 2015 (22%) | 2145 (27%) |
| 65+ | 882 (9%) | 1109 (14%) |
| Blood group |  |  |
| A+ | 2754 (29%) | 2438 (31%) |
| A- | 936 (10%) | 660 (8%) |
| B+ | 666 (7%) | 544 (7%) |
| B- | 274 (3%) | 163 (2%) |
| O+ | 3149 (34%) | 2811 (35%) |
| O- | 1375 (15%) | 1034 (13%) |
| AB+ | 124 (1%) | 233 (3%) |
| AB- | 82 (1%) | 65 (1%) |
| Ethnicity |  |  |
| White | 9126 (99%) | 7812 (98%) |
| Other | 134 (1%) | 136 (2%) |
| Previous low Hb deferrals** |  |  |
| 0 | 8435 (90%) | 7560 (95%) |
| 1 | 791 (8%) | 326 (4%) |
| 2+ | 134 (1%) | 62 (1%) |
| Previous donations** |  |  |
| 0-2 | 3055 (33%) | 1748 (22%) |
| 3-4 | 3695 (39%) | 2723 (34%) |
| 5+ | 2610 (28%) | 3477 (44%) |

*excludes 256 women and 222 men in COMPARE with missing blood group, ethnicity and/or index donation Hb

** in two years preceding index donation. Note in COMPARE, <2% of all donors were new donors with no previous donations ^12^.

**Suppl Table 2 Akaike information criterion for alternative models for time from recall to attendance**

| **Number of knots in flexible parametric rate model** | **Women** | **Men** |
| --- | --- | --- |
| 0-knot (Weibull) | 38,978.53 | 21,825.47 |
| 1-knot | 38,808.63 | 21,680.17 |
| 2-knot | 38,627.17 | 20,337.48 |

Note: lower values of AIC indicate better model fit

**Suppl Table 3 Parameter estimates for time from recall to attendance based on 2-knot flexible parametric model**

| **Parameter** | **HR (95% CI), given return** | |
| --- | --- | --- |
|  | **Women** | **Men** |
| Age category (years) |  |  |
| <25 | 1 | 1 |
| 25 – 34 | 1.12 (1.01, 1.23) | 1.06 (0.93, 1.21) |
| 35 – 44 | 1.08 (0.98, 1.18) | 1.11 (0.98, 1.26) |
| 45 – 54 | 1.03 (0.94, 1.13) | 1.03 (0.92, 1.16) |
| 55 – 64 | 1.05 (0.96, 1.16) | 1.10 (0.97, 1.23) |
| 65+ | 1.07 (0.96, 1.19) | 1.11 (0.98, 1.26) |
| Blood group |  |  |
| A+ | 1 | 1 |
| A- | 1.03 (0.95, 1.11) | 0.97 (0.89, 1.07) |
| B+ | 0.99 (0.90, 1.08) | 1.04 (0.94, 1.14) |
| B- | 0.91 (0.79, 1.03) | 0.88 (0.75, 1.04) |
| O+ | 0.99 (0.93, 1.04) | 0.99 (0.94, 1.05) |
| O- | 0.93 (0.87, 0.99) | 1.00 (0.93, 1.08) |
| AB+ | 1.11 (0.91, 1.35) | 1.11 (0.96, 1.27) |
| AB- | 1.06 (0.84, 1.34) | 1.03 (0.80, 1.32) |
| Ethnicity |  |  |
| White | 1 | 1 |
| Other | 0.96 (0.80, 1.17) | 0.98 (0.81, 1.18) |
| Previous low Hb deferrals* |  |  |
| 0 | 1 | 1 |
| 1 | 1.05 (0.97, 1.14) | 1.19 (1.06, 1.33) |
| 2+ | 1.01 (0.84, 1.21) | 1.11 (0.86, 1.44) |
| Previous donations* |  |  |
| 0-2 | 1 | 1 |
| 3-4 | 1.06 (1.01, 1.12) | 1.09 (1.02, 1.17) |
| 5+ | 1.17 (1.10, 1.24) | 1.34 (1.25, 1.42) |
| Restricted cubic spline 1 | 3.23 (3.16, 3.31) | 3.70 (3.59, 3.82) |
| Restricted cubic spline 2 | 0.92 (0.91, 0.93) | 0.77 (0.76, 0.78) |
| Restricted cubic spline 3 | 0.96 (0.95, 0.96) | 1.21 (1.20, 1.23) |
| Scale | 0.52 (0.43, 0.65) | 0.45 (0.36, 0.56) |

* in two years prior to index donation

**Suppl Table 4 Deferral and dropout parameter values**

|  | **Women** | | **Men** | |
| --- | --- | --- | --- | --- |
| **Transition** | **Observed transitions** | **P(transition)** | **Observed transitions** | **P(transition)** |
| Drop out (non-return within 1y) | 919 / 9360 | 0.0982 | 527/7948 | 0.0663 |
| Non-hb deferral | 461 / 8441 | 0.0546 | 293/7421 | 0.0395 |
| Low hb deferral given below threshold* | 544 / 1467 | 0.3708 | 178/627 | 0.2885 |

* amongst the returners with known Hb at return visit (6547/8441 (78% of women; 5946/7421 (80%) of men)

**Suppl Table 5 Parameter estimates for haemoglobin model**

| **Parameter** | **Coefficient (95% CI), g/dl** | |
| --- | --- | --- |
|  | **Women** | **Men** |
| Age category (years) |  |  |
| <25 | Ref | Ref |
| 25 – 34 | 0.0674 (-0.0185, 0.1534) | -0.0396 (-0.1539, 0.0746) |
| 35 – 44 | -0.0546 (-0.1367, 0.0275) | -0.1823 (-0.2915, -0.0730) |
| 45 – 54 | 0.1116 (0.0328, 0.1904) | -0.2209 (-0.3231, -0.1188) |
| 55 – 64 | 0.1888 (0.1078, 0.2698) | -0.3627 (-0.4653, -0.2601) |
| 65+ | 0.1733 (0.0796, 0.2670) | -0.4945 (-0.6044, -0.3847) |
| Blood group |  |  |
| A+ | Ref | Ref |
| A- | 0.0098 (-0.0607, 0.0803) | 0.0278 (-0.0548, 0.1105) |
| B+ | 0.0963 (0.0155, 0.1771) | 0.1211 (0.0315, 0.2106) |
| B- | 0.0879 (-0.0305, 0.2063) | 0.1500 (-0.00244, 0.3025) |
| O+ | 0.0776 (0.0289, 0.1264) | 0.0815 (0.0294, 0.1336) |
| O- | 0.0182 (-0.0435, 0.0798) | 0.1277 (0.0579, 0.1975) |
| AB+ | 0.0732 (-0.0990, 0.2455) | 0.0946 (-0.0349, 0.2241) |
| AB- | 0.2769 (0.0668, 0.4871) | 0.0866 (-0.1506, 0.3238) |
| Ethnicity |  |  |
| White | Ref | Ref |
| Other | -0.4193 (-0.5841, -0.2546) | -0.3791 (-0.5438, -0.2144) |
| Visit |  |  |
| Index | Ref | Ref |
| Recall | -0.5723 (-0.6700, -0.4746) | -0.4092 (-0.4990, -0.3195) |
| Time since index visit (weeks) | 0.01178 (0.0063, 0.0172) | 0.0038 (-0.0014, 0.0090) |
| Constant | 13.3709 (13.2951, 13.4467) | 15.2794 (15.1798, 15.3790) |

**Suppl Fig 1 Predicted values from linear mixed model (fixed effects only) versus fractional polynomial mixed model in (a) women, (b) men**

**
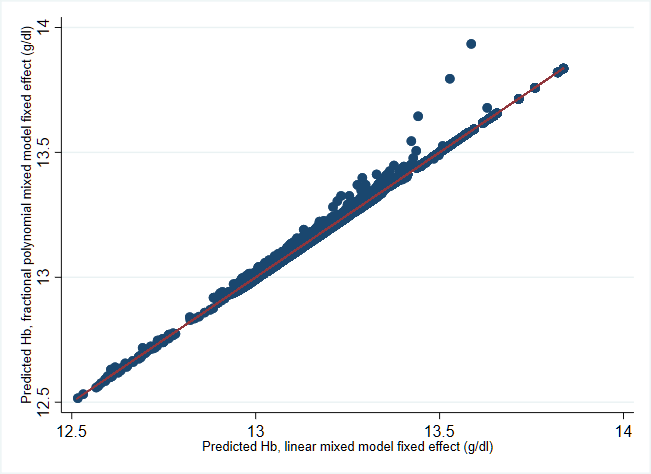
**


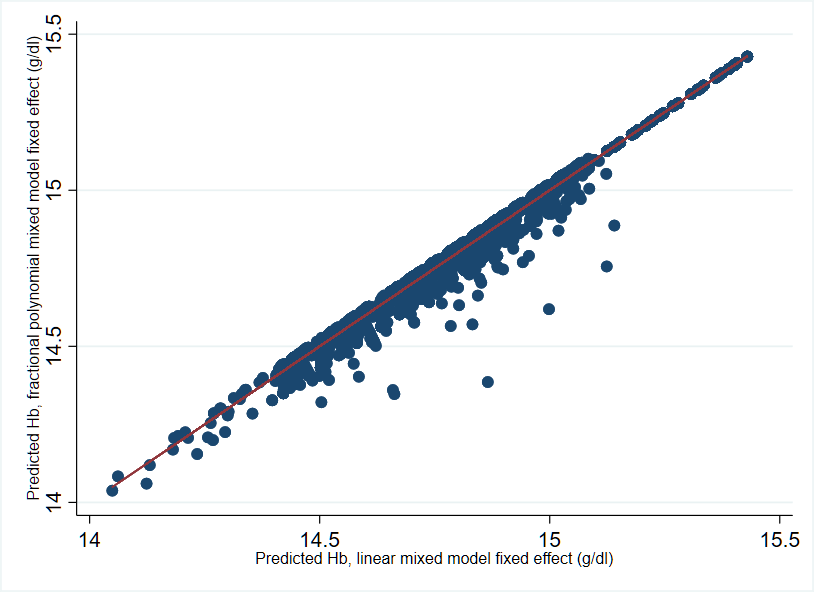


**Suppl Fig 2 Time to return after eligible: Kaplan-Meier and flexible parametric models with 0 (Weibull), 1, and 2 knots in (a) women and (b) men**

(a)

(b)

**Suppl Fig 3 Return times at first return donation visit in women for (a) observed data, (b) DES based on current strategy**

Note: 1562/8441 (19%) of observed returns occur at <16w and are here modelled as 16w returns

(a)


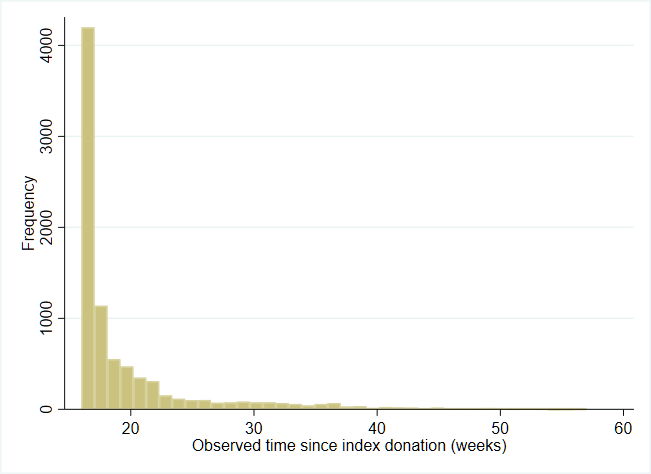


(b)


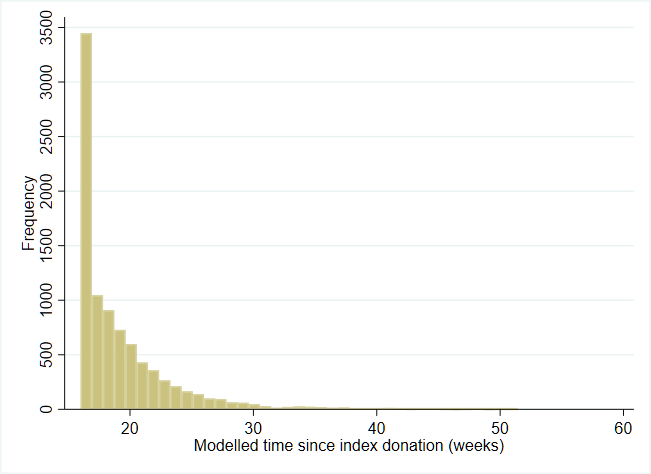


**Suppl Fig 4 Return times at first return donation visit in men for (a) observed data, (b) DES based on current strategy**

(a)


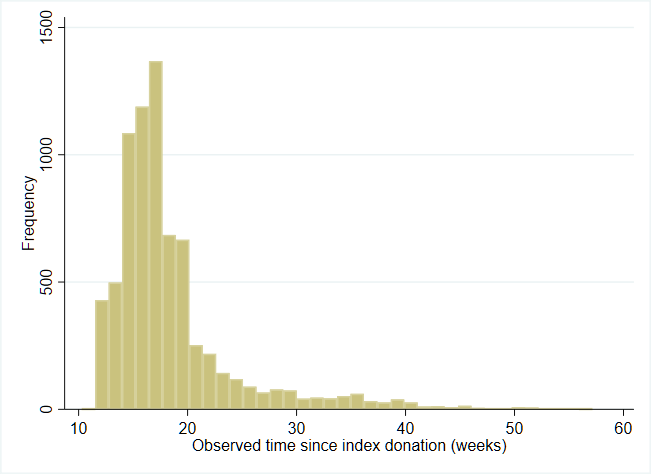


(b)


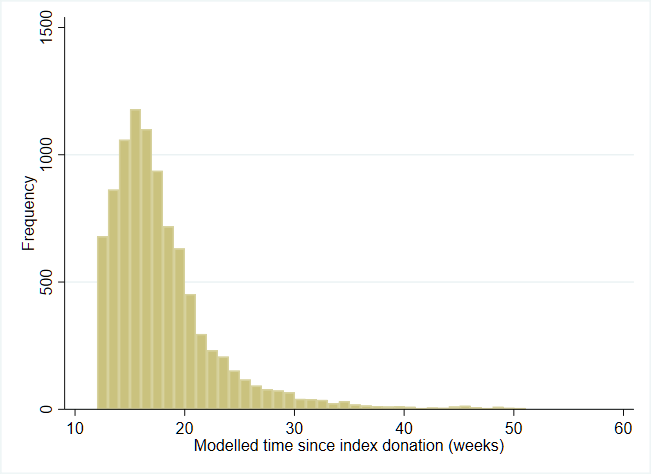


**Suppl Fig 5 Return times at first return donation visit in women for (a) observed data, (b) DES based on current strategy for validation**

Note: 1562/8441 (19%) of observed returns occur at <16w and are included as such here in both the observed and modelled data for validation purposes

(a)


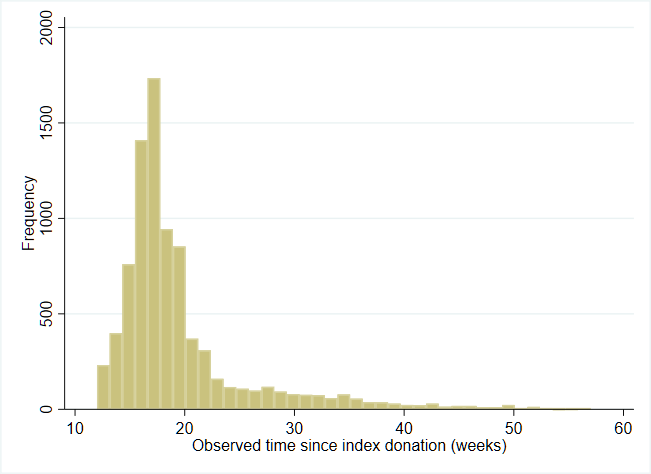


(b)


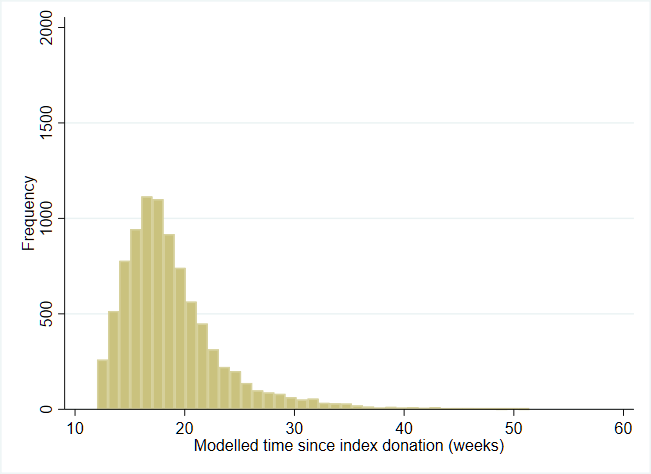


**Suppl Fig 6 Observed versus predicted Hb values at return visit, based on linear mixed model for (a) women, (b) men.** Red points indicate observed over-threshold/predicted under-threshold; purple points indicate observed under-threshold/predicted over-threshold

(a)


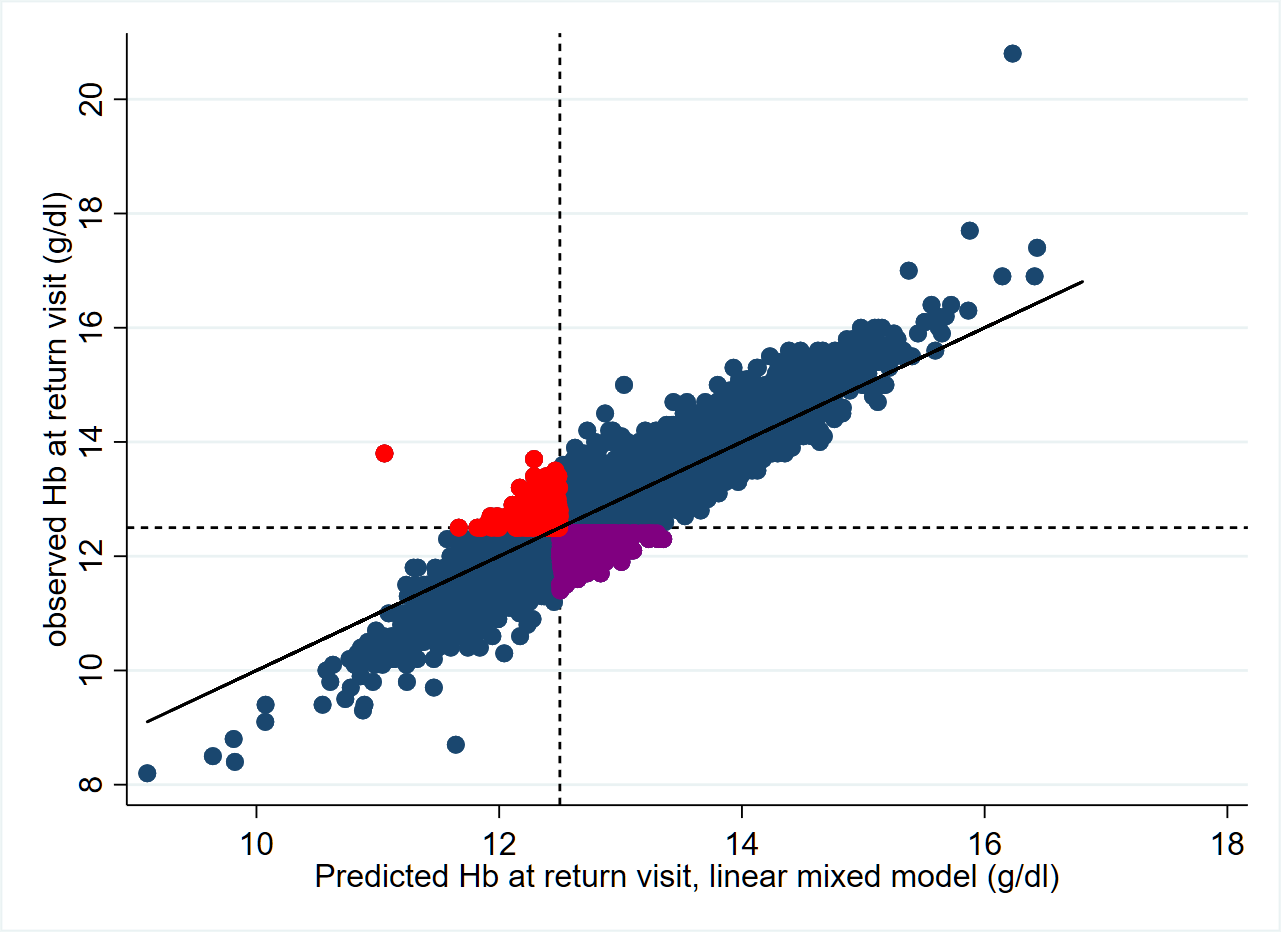


(b)


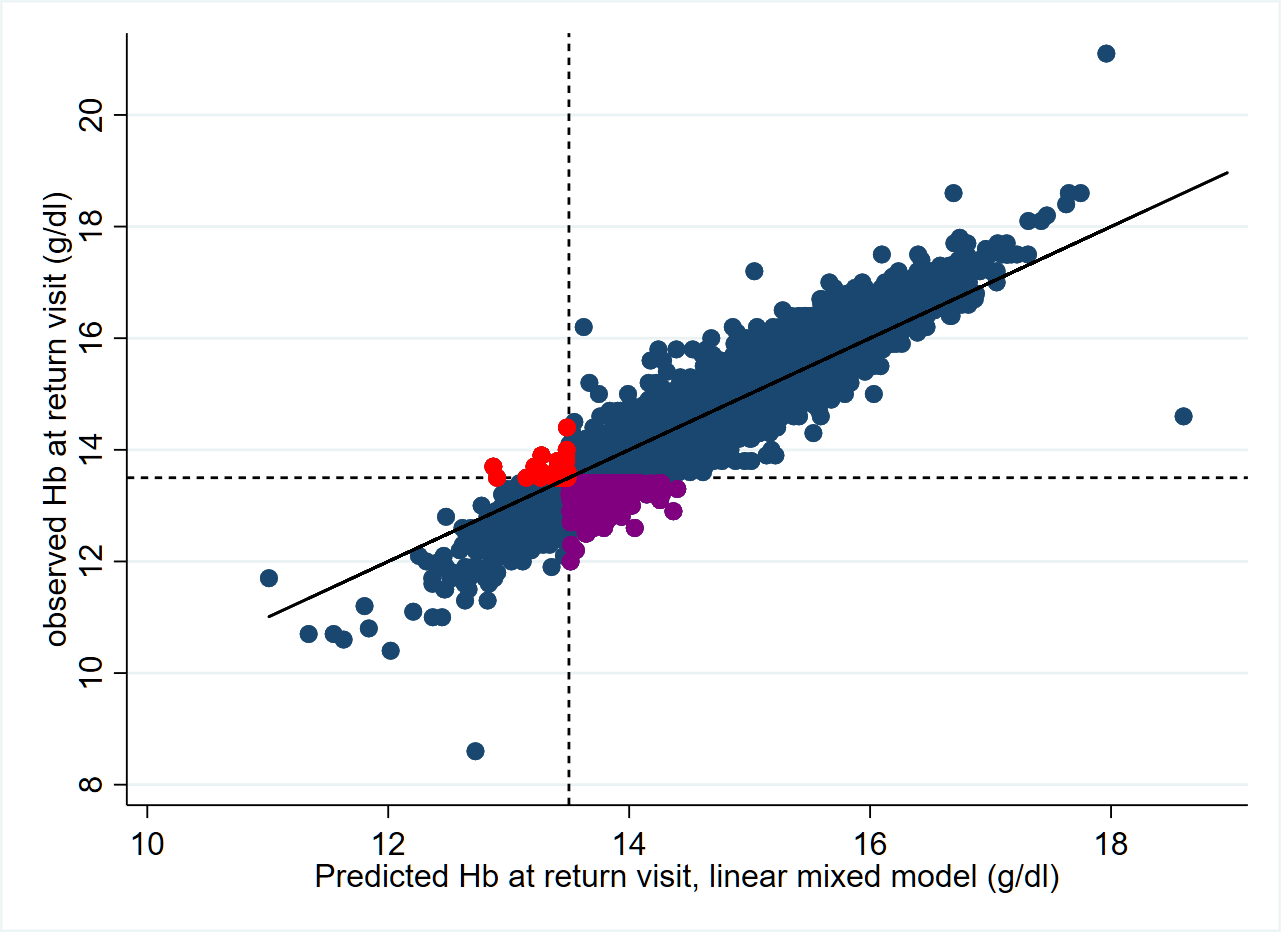

Supplement: Supplementary file 1 — Data S1. Supporting Information [file TRF-63-541-s001.docx]
